# The Science and Art of Midwifery

**Published:** 1892-10

**Authors:** 


THE SCIENCE AND ART OF MIDWIFERY.—By Wm. T. Lusk, A. M., M. D., N. Y. D. Appleton & Co., 1892.

IT IS not necesssry to go into a strict review of a book so well known to the profession as this work of Dr. Lusk's, which now appears in its fourth edition.

The author has revised some of the principles taught in the former editions, thus bringing the work fully abreast with the latest researches on all subjects pertaining to the subject-matter. He heartily endorses strict adherence to aseptic and antiseptic midwifery as carried out in the principles of modern surgery, without any of the pedantic measures that have proven useless.

Chapter XVII. is devoted entirely to the subject of extra-uterine pregnancy and deserves a careful perusul by all who feel that they are not conversant with this subject that has of late attracted so much attention.

The book is neatly gotten up and deserves a place in every library. W. A. C.

